# A Qualitative Assessment of the Risk of Introducing Peste des Petits Ruminants into Northern Zambia from Tanzania

**DOI:** 10.1155/2014/202618

**Published:** 2014-01-12

**Authors:** R. Chazya, J. B. Muma, K. K. Mwacalimba, E. Karimuribo, E. Mkandawire, M. Simuunza

**Affiliations:** ^1^Department of Veterinary Services, Ministry of Agriculture and Livestock, P.O. Box 830011, Mumbwa, Zambia; ^2^Department of Disease Control, School of Veterinary Medicine, University of Zambia, P.O. Box 32379, Lusaka, Zambia; ^3^Independent Policy Researcher, 4639 D Santa Cruz Drive, Indianapolis, IN 46268, USA; ^4^Department of Veterinary Medicine and Public Health, Sokoine University of Agriculture, P.O. Box 3021, Morogoro, Tanzania

## Abstract

A qualitative risk assessment was performed to evaluate the risk of introducing Peste des petits ruminants virus into northern Zambia from Tanzania via live goat trade. Data was collected during a mission to Tanzania and northern Zambia and also from literature and interviews with experts. The risk of PPRV introduction was evaluated as a function of the probability of hazard (PPRV) release, exposure of susceptible hosts, and the consequences of spread using the following parameters: prevalence of infection, volume of trade, C-ELISA and quarantine screening missing an infected animal, PPRV viability (remaining infective) in transit, and the virus potential for infection. The magnitude of the consequences was derived from the probability of transmission and spread and the impact of PPRV introduction and establishment. Accordingly, the probability of occurrence of PPRV in northern Zambia from Tanzania was rated as “high” and the economic consequences were also rated as “high.” Finally, the overall risk of introducing PPRV into northern Zambia from Tanzania at the time of the assessment was rated “high.” It was concluded that import of goats and sheep be prohibited until efficient and adequate measures to reduce the risk have been put in place.

## 1. Introduction

Peste des petits ruminants (PPR) is a viral disease of small ruminants that threatens the national food security of affected countries. The disease has high morbidity and mortality rates and greatly impacts the economies of affected countries, often as a consequence of trade loss due to sanitary embargoes [1]. In the Southern African Development Community (SADC) region, the disease was officially reported in northern Tanzania in 2008 [2] and later spread to the south in 2010 [3]. The Democratic Republic of Congo (DRC) reported outbreaks of PPR that caused death of almost 120,000 small ruminants, valued at US$5.3 million from 2010 to June 2012 [4]. This did not take into account the socioeconomic and other benefits of goats and sheep to smallholder farmers [5]. In October, 2012, Angola reported an outbreak of PPR in Cabinda Province due to illegal movement of a herd of 55 sheep and goats brought from DRC [6]. Therefore, PPR is a disease of increasing importance in Africa, particularly in areas where small ruminants form an important component of agricultural food production. Livestock production is an important agricultural activity in most of the countries in Southern Africa [7] and goats play an important socioeconomic role in rural areas, especially for women who are among the most vulnerable farmers in Africa. This is because goats and sheep are prolific and require low capital inputs for a moderate level of production, reaching maturity early and are profitable to keep [8]. Until recently, PPR was considered absent in the SADC region, and as such most countries have not developed strategies on how to stem the spread of the disease in the event of an incursion [9]. It is therefore assumed that, if the disease were allowed to spread from the DRC and Tanzania into the whole of the 15-nation SADC region, it could potentially devastate the livelihoods and food security of millions of vulnerable smallholder farmers and agropastoralists [9]. In Zambia, PPR is a notifiable disease which has not been reported despite its sharing borders with the affected countries [5].

PPR is caused by the PPR virus (PPRV) which belongs to the genus *morbillivirus* under the family Paramyxoviridae [1]. PPRV is an RNA virus, which is closely related to the measles, rinderpest, and distemper viruses [10]. There is only one serotype of PPRV, but there are at least 4 lineages which are distinguishable by nucleic acid sequencing. The virus is not very resistant and is rapidly inactivated at environmental temperatures by solar radiation and desiccation [10]. Transmission of PPR between infected and susceptible hosts is achieved by direct contact, close contact, or through respiratory and oral routes [11]. PPRV targets epithelial cells and pneumocytes leading to respiratory lesions including interstitial pneumonia and bacterial bronchopneumonia [12] as well as bronchointerstitial pneumonia [13]. The lymph nodes are characterized by oedema [13]. PPR is characterized by high fever, erosive stomatitis, mucopurulent nasal and ocular discharge, pneumonia, necrosis and ulceration of mucous membranes, and inflammation of gastrointestinal tract leading to severe diarrhoea [14]. The PPRV is biologically and antigenically related to rinderpest virus and, clinically, the disease mimics rinderpest in goats [15]. Clinical disease is seen in sheep and goats and has been described in zoological garden collections of wild small ruminants including Laristan sheep (*Ovis gmelini laristanica*), Dorcas-type gazelles (*Gazella granti*), gemsbok (*Oryx gazelle*), and the Nubian ibex (*Capra ibex nubiana*). Cattle, buffaloes, camels and pigs can become infected but there is little or no evidence of disease associated with their infection [16–18]. PPRV antigen has been detected in an outbreak of respiratory disease in camel and sick domestic buffaloes [19–21]. The tenacity of PPRV is considered to be low as the virus does not usually remain infective outside the body for longer than four days given the usual climatic conditions for Southern Africa. The virus is inactivated by UV light and most lipid-solvent based detergents and is both thermo- (>70°C) and pH-labile (inactivated at pH < 5.6 and >9.6) [18]. It is not well understood how the virus is maintained between outbreaks [18]. PPR outbreaks have been associated with seasonal variations, that is, more frequent outbreaks occur during the rainy season or the dry cold season. PPR is also associated with seasonal periods of increased local trade in goats [17].

The ruminant livestock subsector in Zambia contributes about 35% to the national agricultural output. The country has a livestock population estimated at 3.6 million cattle, 0.6 million sheep, 1.8 million goats, 33 million poultry, and 1.1 million pigs [22]. The Zambian livestock sector plays an important role in socioeconomic development, household food and nutritional security, and poverty alleviation. It accounts for about 36.4% of total agricultural production. About 23% of the per capita supply of protein comes from animal products [22]. Trade in livestock and livestock products exist between Tanzania and Zambia. Despite the imminent risk of PPR spreading from Tanzania into northern Zambia, there has been no objective risk assessment conducted to determine the risk of pathogen introduction into the country through importation of livestock commodities from Tanzania into Zambia. This paper therefore reports the results of a qualitative assessment of the risk of introducing PPR virus from Tanzania into northern Zambia based on the data collected in 2012.

## 2. Materials and Methods

### 2.1. Study Areas

**Figure 1:**
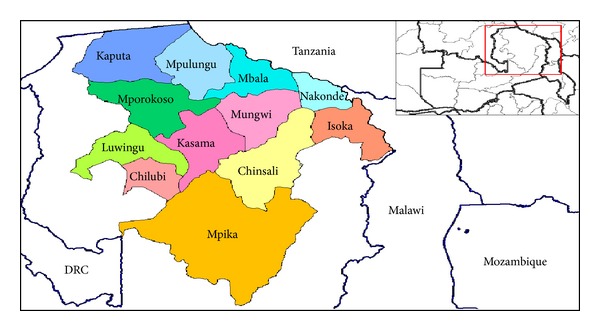
Location of Mpulungu, Mbala, and Nakonde in relation to other districts of Northern Zambia and Tanzania on North West. Tanzania is located in the north.

The study was conducted in 3 districts of northern Zambia, namely, Mbala, Mpulungu, and Nakonde, in 2012 (Figure 1). The districts were purposively selected based on their relative proximity to Tanzania and human and animal traffic between Zambia and Tanzania. The geographical position of Mbala is latitude 8°50.4144′ South and longitude 31°21.9522′ East, that of Mpulungu is latitude 8°46.0002′ South and longitude 31°7.9998′ East, while that of Nakonde is latitude 9°20.5278′ South and longitude 32°44.7′ East. The total goat and sheep population in the three districts is estimated at 36,662 and 1,148, respectively [23].

### 2.2. Data Collection and Tools

Data used to estimate the model input parameters was collected during a study visit to Tanzania from 17 to 24 October, 2012, where discussions were held with officials from the National Epidemiological Unit in the Ministry of Livestock and Fisheries Development (MLFD); relevant documents were obtained, and linkages to other officers involved in PPR control were made. Data was collected on disease prevalence, disease distribution, control measures, livestock movement patterns between Zambia and Tanzania, PPR vaccination coverage, and other relevant epidemiological and surveillance data.

Further data was collected through a questionnaire survey conducted along the border areas of Zambia and Tanzania from 10 to 24 October, 2012. The areas included Sumbawanga and Tunduma districts in Tanzania and Mbala, Mpulungu, and Nakonde districts of Zambia. Livestock farmers, veterinary staff, border staff, and other stakeholders were interviewed. A structured questionnaire with different sections for farmers/traders, veterinary staff, and border staff was used. Purposive sampling was employed to select initial respondents, and snowball sampling was used to identify subsequent interviewees with the help of area veterinary assistants. Questionnaires were administered on one-on-one basis with the interviewer sitting down with the respondents. One hundred and thirty-eight farmers, twelve veterinary staff working in border areas, and three border staff were interviewed using local languages (Mambwe, Namwanga, and Bemba) or English where appropriate. In-depth oral interviews were also conducted with district veterinary officers (DVOs) for Nakonde, Mbala, and Mpulungu with a focus on gathering data on livestock populations: common diseases in goats and sheep, husbandry practices by farmers, movement patterns of livestock between the two countries, surveillance methods used, knowledge on PPR, and capacity of the veterinary department to conduct surveillance.

Data from the questionnaire was coded and entered into a spreadsheet using Microsoft Excel. Data obtained from oral interviews was transcribed and transferred into Microsoft Word for further analysis. GIS coordinates were entered into a spreadsheet using Microsoft Excel. Other sources of information were from published literature, grey literature, on-line publications through internet searches (key terms: PPR risk*, PPR surveillance* PPR prevalence* PPR control*, PPR policy* Peste des petits ruminants* and specific databases such as pubmed and Google scholar) and expert opinions.

### 2.3. Qualitative Assessment

The methods used to conduct this risk assessment were based on the World Organisation for Animal Health (OIE) Terrestrial Animal Health Code 2001 framework and the work of Zepeda [24]. Evaluation of the following factors, both in the country of origin (Tanzania) and destination (Zambia), was also conducted: organisation of the veterinary structure; presence and capability of diagnostic facilities; epidemiological surveillance systems; disease status in Northern Province; animal population and movements; and the legal framework.

#### 2.3.1. OIE Risk Assessment Framework

For import of animals and their products, the technical steps in a risk analysis as per [25] guidelines were done as follows: (a) frame the question, (b) identify the hazards, (c) model the pathway (outline the conceptual model and develop the risk scenario trees), (d) collect information (quantify inputs and impacts; probability combinations), (e) assess the risk (release assessment, exposure assessment, consequence assessment and risk assessment), and (f) describe uncertainties in the qualitative model.

#### 2.3.2. The Risk Question

The question we were attempting to answer is “what is the annual risk of introducing PPR virus into northern Zambia through importation of live goats from Tanzania?”

#### 2.3.3. Hazard Identification

The hazard identified is the morbillivirus causing Peste des petits ruminants (PPR) in goats and sheep.

#### 2.3.4. Risk Scenario Trees

**Figure 2:**
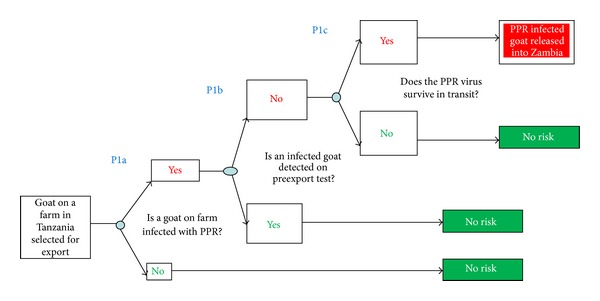
Scenario tree for PPR exposure to goats in Northern Zambia.

**Figure 3:**
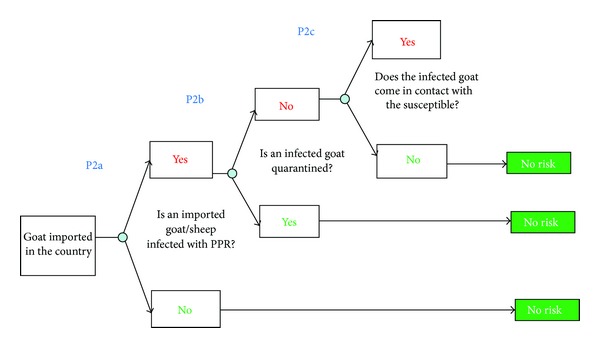
Scenario tree for PPR consequence of release, exposure, and infection.

**Figure 4:**
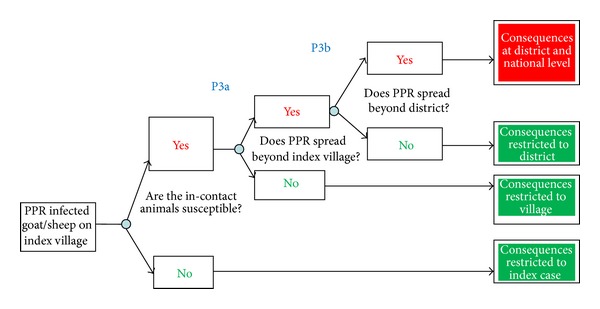
Scenario tree for consequence assessment.

The scenario trees for release of PPR into Northern Province of Zambia, exposure assessment, and consequence assessment are shown in Figures 2, 3, and 4.

In this study, the probability of occurrence of the risk (PPR virus infection and the consequences of the outbreaks) was equated to the probability of entry of the hazard (from Tanzania to northern Zambia) combined with the probability of exposure of susceptible animals to PPR virus. Each parameter in the assessment was evaluated based on the available information [24]. The descriptive scale developed by Zepeda [24] was used to evaluate the probability of occurrence of each event (Table 1). Combination of probabilities at each stage of the pathway, that is, release, exposure, and consequence, was done using a combination matrix (Table 2).

## 3. Results

### 3.1. Probability of Release

The four parameters examined in order to determine the probability of release of the PPRV into northern Zambia from Tanzania were as follows: probability of PPR virus infection of a goat selected for export; volume of trade; probability of C-ELISA and quarantine screening missing an infected goat; probability of pathogen viability (remaining infective) during transit.

#### 3.1.1. Probability of Infection

The probability of infection of a goat selected for export is a function of the probability of occurrence of the hazard. It was dependent on the following factors: prevalence of PPRV from the area of origin; organisation and efficacy of the veterinary epidemiological surveillance system; diagnostic facilities; and PPR vaccination coverage.

A serosurvey carried out in northern Tanzania (Ngorongoro, Monduli, Longido, Karatu, Mbulu, Siha and Simanjiro) indicated an overall seroprevalence of PPRV infection in small ruminants of 45.8%. Highest seroprevalence (42.6–88.02%) was observed in Mbulu, Siha, Longido, and Ngorongoro districts [26]. Laboratory findings confirmed presence of PPRV in southern Tanzania by RT-PCR and serological analysis revealed that seroprevalence was 31% [27]. The official veterinary network in both countries, that is, Tanzania and Zambia is well structured and covers the whole country in terms of territory. In Zambia, districts are divided into veterinary camps run by veterinary assistants (VA's) who in turn report to district veterinary officers (DVOs). In Tanzania, the equivalent of a veterinary camp is a ward which is subdivided into villages. Wards are run by livestock field officers (LFOs) who in turn report to DVOs. DVOs run districts and are in charge of all disease control activities in their territories. However, in Tanzania, district vets are under the local government, hence at times it is difficult for the Department of Veterinary Services (DVS) to get information from them. For example, in 2009 during the PPRV outbreak, it took almost 1 year from suspicion to disease confirmation [28]. This clearly presents challenges in disease surveillance.

In Zambia, there are VAs on the ground but not all veterinary camps are manned. A number of districts face this challenge which impacts negatively on disease surveillance and control activities. Moreover, compared to agricultural camps, veterinary camps are too large for one VA to effectively manage. For example, in the study area, Mpulungu District only had one veterinary camp against 14 agricultural camps for the same territory. One VA in Mpulungu covered a radius of more than 75 Km, the furthest distance being 204 Km within the district. The staffing situation of VAs in veterinary camps was severely inadequate.

The MLFD in Tanzania had seven zonal laboratories and one central laboratory. Capacity of the laboratory staff to diagnose PPR was good as most laboratory staff were trained. Three regional laboratories and one central laboratory were able to diagnose PPR using ELISA. Recently, the central laboratory had acquired capacity to conduct molecular diagnosis of PPRV. The antibody-based C-ELISA kits, the common test that was used, were acquired from the United Kingdom though at times they faced some problems acquiring them.

Zambia has five regional laboratories, namely, Chipata, Mazabuka, Ndola, Mongu, and Isoka and one central veterinary laboratory. The regional laboratory in northern Zambia (Isoka) was nonfunctional. Out of all these laboratories, only the Central Veterinary Research Institute (CVRI) had capacity to diagnose PPR. CVRI had 4 members of staff who had been trained in PPR diagnosis using C-ELISA. The C-ELISA is used to detect presence of antibodies to PPRV in the serum by quantifying the amount of monoclonal antibody (MAb) [29]. Most commonly used ELISA kits for PPR-specific diagnosis show that, sometimes, it does not detect PPR virus antigens from some clinical samples and B95a cell derived virus. Sandwich ELISA has been developed which is able to detect PPRV antigens from clinical samples [29]. The test has undergone extensive field evaluation and has been found to be suitable for the diagnosis of acute PPR virus infection [29]. However, this test is not available in Tanzania.

Although farmers in northern Tanzania were aware of efforts being made to control the disease, only 32% had their animals vaccinated against PPR [26]. The low vaccination coverage suggests continued presence of PPR in the study area. It was concluded that there was limited capacity with respect to veterinary disease surveillance, reporting and control of transboundary, and emerging diseases which needed to be addressed in the country [26].

Based on the preceding information, that is, seroprevalence of the disease in Tanzania of 45.8% [26] and 31% [29], a low vaccination coverage in high-risk areas [28], reasonable but inadequate diagnostic capability from the state laboratories, and well-organized veterinary structure covering the whole country, the probability that any one animal selected for export would be infected with PPR virus was determined to be *high*.

#### 3.1.2. Volume of Trade

The questionnaire survey conducted along the border areas between Zambia and Tanzania indicated the following:

1. on average, each farmer purchased and brought (from different sources) into the flock 2 (95% CI: 1–3) goats in a year;
2. forty-eight farmers bought at least one goat in the last 1 year (215 goats in total);
3. goat sources: 95.6% (CI: 92.2–99.0) bought within the village, 2.2% (0–4.6) bought within district, and 1.4% (0–3.5) bought within the province;
4. twenty-two goats were purchased from Tanzania by 17 farmers among those interviewed; on average each farmer bought 1 goat in a year;
5. an interview of the 11 veterinary staff working on the border areas estimated that 4,612 goats (95% CI: 2,296–11,520) were imported into the country in the last 1 year.

Based on the above considerations, the probability of entry as determined by trade volume was rated *low*.

#### 3.1.3. Probability of C-ELISA and Quarantine Screening Missing an Infected Goat

C-ELISA has a relative specificity of 98.4% and a relative sensitivity of 92.4% [30]. Sensitivity of C-ELISA for PPR serosurveillance could further be increased to 95.4% if the target population is nonvaccinated [30]. There were 19 quarantine facilities for farm animals and 28 wildlife quarantine facilities under supervision of Veterinary Services in Tanzania (Zoosanitary inspectorate Services, MLFD). Field staff in Tanzania was able to diagnose clinical cases of PPR [28]. This implied that an infected animal which manifested clinical signs of PPR during quarantine could be diagnosed and removed from the consignment and destroyed. Choi et al. [31] reported a relative specificity and sensitivity of the rapid C-ELISA of 98.5% and 93.4%, respectively. Therefore, the probability that both C-ELISA and quarantine screening will miss an infected goat on preexport screening was determined to be *negligible*. However, trade in livestock between the two countries was primarily informal, hence most animals were not likely to go through quarantine and screening, and hence this assessment may underestimate the risk for such type of movements.

#### 3.1.4. Viability of the Pathogen (Remaining Infective) during Transit

Tears, nasal discharges, coughed secretions, and all other secretions and excretions of incubating and sick animals are all sources of the virus [32]. Due to an incubation period of 4-5 days and considering the travel period between Tanzania and Zambia (1-2 days), an infected goat will be able to carry the virus over long distances and be able to shed it to in-contact susceptible animals. Based on the evidence that suggests viability of the pathogen in live goats, and considering the short distance travelled across the border from Tanzania, the probability that the virus would remain viable during transit was *high*.

#### 3.1.5. Assessment

Using the matrix (Table 2), the probability of release is a function of the combination of risks relating to probability of infection (high), volume of trade (low), C-ELISA and postexport quarantine screening missing a positive animal (negligible), and viability of pathogen (high); thus, the probability of release is rated “moderate” (Figure 1).

### 3.2. Probability of Exposure

The parameters used to determine the probability of exposure were as follows: the probability of an imported goat/sheep being quarantined (posttransit); probability of a positive animal having contact with susceptible goats or sheep on index village; proportion of susceptible contact goats and sheep; probability of transmission of PPR virus to susceptible goats.

#### 3.2.1. Probability of the Posttransit Quarantine Missing a Positive Animal

An in-depth interview with the DVOs for Mbala, Mpulungu, and Nakonde revealed that there were no quarantine facilities in the 3 districts, instead on-farm quarantine was employed when need arose. The herd/flock or individual animals were placed under observation for a period of not less than 21 days. The animals were monitored by a VA for development of clinical signs/disease under the supervision of the DVO, who authorized the end of the quarantine after certifying for disease absence. The challenge with this type of quarantine was that properly fenced quarantine facilities that can adequately restrict animal movement were nonexistent. Monitoring was also infrequent. The Veterinary Department did not always provide resources at camp level to do this effectively, including surveillance. Hence the camp officers relied on farmer compliance. There was a clear possibility that mixing with local animals could still occur even when animals were under quarantine.

An interview with the officer responsible for Zoosanitary Inspectorate unit of the Ministry of Livestock and Fisheries (MLFD) in Tanzania revealed that there was little formal trade/export of live animals from Tanzania into Zambia. This meant that much trade relating to livestock between the two countries was informal or illegal. This situation made it difficult to carry out quarantine and inspection of imported goats/sheep into Zambia from Tanzania. On-farm quarantine under supervision of veterinary services for formal movements remained the only option, while informal movement's was a challenge. This quarantine was only employed in situations where the Veterinary Department had information of illegal movement of livestock. Based on the evidence that suggested lack of quarantine facilities; illegal animal movements; and on-farm quarantine that was unsecure (no fences) and poorly monitored, the probability of post-transit quarantine screening missing an infected animal was *high*.

#### 3.2.2. Probability of Contact with Goats/Sheep in Index Village

PPRV is considered to be highly infectious, often spreading rapidly between groups of susceptible animals [18]. Infection may be associated with: the introduction of animals from another area; the general movement of animals; contact with livestock returning unsold from market; contact with traded livestock or nomadic animals (e.g., shared grazing land, water, housing); and husbandry changes [18].

The questionnaire survey with farmers and traders in northern Zambia revealed the following as sources of goats: 95.6% (95% CI: 92.2−99.0%) bought goats within the village; 2.2% (95% CI: 0−4.6%) within the district, and 1.4% (95% CI: 0 to 3.5%) within the province. As to whether the imported goats were mixed with local goats, 61.8% (95% CI: 51.3 to 72.5%) of 84 respondents indicated that the goats bought from Tanzania did not mix with the local goats while 38.1% (95%, CI: 27.5 to 48.7%) indicated knowledge of mixing. Separate interviews with 11 veterinary staff working on the border areas estimated that 4,612 goats (95% CI: 2,296 to 11,520) were imported into the country in the last 1 year. However, most of these goats were imported into Nakonde and were slaughtered in restaurants. For instance, one restaurant owner indicated slaughtering 8 goats per day. This significantly contributed to reducing the risk of contact with susceptible goats. Thus, the risk of PPRV transmission from imported goats to susceptible animals in northern Zambia was considered to be *low*.

#### 3.2.3. Proportion of Susceptible Contact Goats/Sheep

There have never been reported outbreaks of PPR in goats and sheep in Northern Province of Zambia. Therefore, small ruminant populations will be expected to be naïve to PPR virus infection. In Northern Province of Zambia, goats were kept under free range and chances of exposure to the virus from infected goats and sheep were likely to be high in such type of husbandry practice. Moreover, vaccination of small ruminants against the disease was not practiced; hence the animals had no immunity to the PPRV. We therefore determined that the proportion of susceptible goats in the recipient flock was *high*.

#### 3.2.4. Probability of Transmission of PPR Virus to Susceptible Goats/Sheep

PPR is a severe and fast-spreading, highly contagious and infectious viral disease of domestic and wild small ruminants [17]. Transmission of PPR is achieved by direct contact from infected to susceptible animals by close contact or through respiratory and oral routes [11]. Up to 100% of the animals in a flock may be affected in a PPR outbreak with between 20 and 90% mortality [17]. We therefore determined the probability of transmission to be *high*.

#### 3.2.5. Assessment

Using the matrix (Table 2), the probability of exposure is a function of the combination of risks relating to probability of the post transit quarantine missing an infected animal (high), probability of contact with goats/sheep in index village (low), proportion of susceptible contact goats (high), and probability of transmission of PPR virus to susceptible goats (high); thus, the probability of exposure was rated “*High*” (Figure 1).

### 3.3. Magnitude of the Consequences

In the border districts of Northern Province, goats are mainly kept by traditional farmers on a very small scale. Local breeds of cattle with no genetic improvement are thus kept. However, morbidity and mortality rates in susceptible naïve populations are high. Morbidity of up to 100% and mortality rates between 20 and 90% are common, except in endemic areas or when mild disease occurs [10, 33].

While the direct consequences may be marginal, indirect ones are likely to have far reaching effects. Presence of PPRV in the area may not only affect trade in small ruminants from these areas but also the cattle and the pig industries in the area are likely to suffer from trade restrictions in case of an outbreak. There is significant livestock trade in Northern Province to warrant concern over PPR incursions. Once the disease is in northern Zambia, the rest of the county could easily get infected considering the limited disease surveillance efforts in place and inadequate veterinary service delivery. Such an outbreak may be challenging to control considering there are no emergency preparedness plans in place to combat such an outbreak in the country, although at regional level, SADC could be expected to render some assistance. Therefore, the whole of Zambia could easily suffer from a ban on export of live animals or animal products. In this context, the possible impacts of PPRV outbreak were considered “*High*”.

### 3.4. Probability of Occurrence

**Figure 5:**
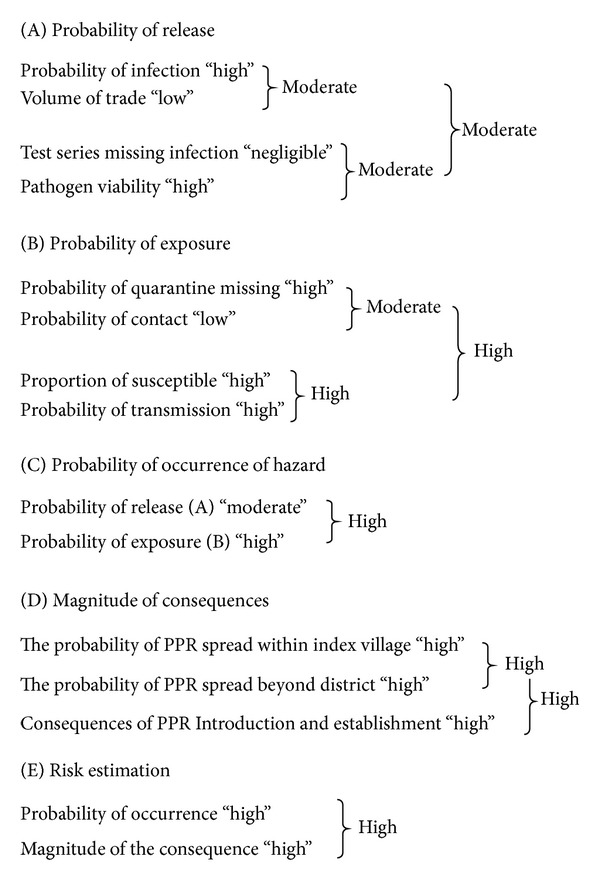
The risk estimation process.

Probability of occurrence is a product of two probabilities; the probability of release/entry (P1) which was rated *moderate* and the probability of exposure (P2) which was rated *high*. Based on the combination matrix of probabilities employed in this study (Table 2), the probability of occurrence was thus *high* (Figure 5).

### 3.5. The Assessed Risk of PPRV Introduction into Northern Zambia

The assessed risk is a combination of the probability of occurrence (*high*) and the magnitude of the consequences of occurrence (*high*), and is thus rated “*high*”.

From the interpretation of the probability scale provided by Zepeda Sein (Table 1), high implies prohibiting import until measures to reduce the risk have proven their efficiency and adequate verification procedures are available to ensure safe implementation. All uncertainties in the qualitative model were described (Table 3).

## 4. Discussion

In this paper, we report the results of a qualitative assessment of the risk of introducing PPR virus from Tanzania into northern Zambia based on the data collected in 2012. Our design was based on evaluating the risk of PPR virus introduction through formal trade in goats. However, the study revealed that most movements of goats were informal, and we thus redesigned the investigation plan to take these into account in assessing the risk. Under informal trade, little information is captured by government officers and therefore the information provided by the veterinary and border staff was likely to underestimate the actual trade volumes and other investigated parameters. It is therefore acknowledged that there are uncertainties surrounding the probability estimates in our study, especially data from government officials.

The probability of occurrence of PPR virus in Northern Province of Zambia was determined to be *high*. The magnitude of the consequences of occurrence of PPRV was also determined to be high. The assessed risk was a combination of the probability of occurrence (*high*) and the magnitude of the consequences of occurrence (*high*), and was thus rated “*high*”. From the interpretation of the probability scale provided by Zepeda et al. [24], high implies prohibit import until measures to reduce the risk have proven their efficiency and adequate verification procedures are available to ensure safe implementation. Although prohibition of import could be recommended, its implementation would be difficult since much trade in goats and sheep between Zambia and Tanzania was informal.

Therefore, risk management measures could include formalising trade in small ruminants between the two countries and strengthening veterinary services and capacity to conduct epidemiological surveillance. Since most of the cross border movements of goats and sheep were informal, there is need for the Department of Veterinary Services along the border on both sides to create awareness among the farmers of the need for government to regulate trade in livestock and also intensify surveillance, monitoring, and livestock movement controls. A joint surveillance system by both Tanzanian and Zambian Veterinary authorities could be put in place. This could help prevent the spread of transboundary diseases between the two countries, including PPRV.

Another important measure of mitigating the risk is to create a vaccination buffer/zone of 50 km from the border into the high-risk areas to avert a possible incursion of PPR [5]. PPR vaccine has been proved to be protective to small ruminants for a period of at least 3 years [17]; hence most animals will only need vaccination twice in their lifetime. Subsequent vaccinations should target naïve newly born kids/lambs. At least 85% of the small ruminant population should be vaccinated in the high-risk zone; this includes Mbala, Nakonde, Mpulungu, and possibly Isoka. If the strategy of vaccinations is taken up, there will be need to build laboratory capacity to carry out the differentiation of infection from vaccinated (DIVA) animals. In the absence of this, proper animal identification will be required, for example, ear tagging or branding of all vaccinated animals.

## 5. Conclusion

Considering that northern Zambia has a small population of goats and sheep, the actual consequences resulting from losses in terms of mortality and morbidity are likely to be minimal. However, the indirect losses far outweigh the direct losses and these should prompt the veterinary Department to implement measures to prevent any such incursions.

## Figures and Tables

**Table 1:** Practical interpretation of qualitative probability ratings [24].

Term	Meaning for probability of event occurrence	Meaning for consequence	Meaning for risk estimated
Negligible	Probability of occurrence of the event is possible only in exceptional circumstances	Low or no impact	Allow import without restrictions
Low	Occurrence of an event is a possibility in some cases	Minor impact	Authorise with specific measures to reduce the risks
Moderate	Occurrence of the event is a possibility	Average impact	Provide assessment of mitigation options before authorising
High	Occurrence of the event is clearly a possibility	Serious impact	Prohibited until measures to reduce the risk have proven their efficiency (implement and audit)

**Table 2:** Matrix showing probabilities when two parameters are combined [24].

Results of the assessment of Parameter 1	Results of the assessment of Parameter 2			
	Negligible	Low	Moderate	High
Negligible	Negligible	Low	Low	Moderate
Low	Low	Low	Moderate	Moderate
Moderate	Low	Moderate	Moderate	High
High	Moderate	Moderate	High	High

**Table 3:** Description of model uncertainties.

Assessment component		Uncertainty description	Direction of error	Magnitude
Release	Probability of C-ELISA and quarantine missing an infected goat	There have been no formal movement of goats/sheep from Tanzania into Zambia, hence most goats will not undergo testing and quarantine	Underestimated risk	High
	Volume of trade	Most movements are illegal due to the porous border resulting in many trade routes	Underestimated risk	Moderate

Exposure	The probability of the quarantine missing a positive animal	There are no known quarantine stations in Northern Zambia. On-farm quarantine is difficult to enforce in cases where illegal animal movements are not detected	Underestimated risk	High

## References

[1] Wambura PN (2000). Serological evidence of the absence of peste des petits ruminants in Tanzania. *Veterinary Record*.

[2] Swai ES, Kapaga A, Kivaria F, Tinuga D, Joshua G, Sanka P (2009). Prevalence and distribution of Peste des petits ruminants virus antibodies in various districts of Tanzania. *Veterinary Research Communications*.

[3] Decapua J Peste des petits ruminants in Southern Tanzania. http://coalgeology.com/deadly-animal-virus-peste-des-petits-ruminantsthreatenstospread-to-southernAfrica/8302.

[4] FAO (2013). *Livestock Epidemic Causing Havoc in Democratic Republic of the Congo*.

[5] SADC (2012). *SADC Control Strategy for Pest de Pestes Ruminants*.

[6] OIE (2012). Peste des petits Ruminants [PPR]. *Weekly Disease Information*.

[7] Mamabolo MJ, Webb EC (2005). *Goat Production Survey-Fundamental Aspects to Model Goat Production Systems in Southern Africa-Case Study*.

[8] Devendra C, Burns M (1970). *Goat Production in the Tropics*.

[9] SADC (2012). *SADC Pestes des Petits Ruminants (PPR) Control and Eradication Working Group*.

[10] Geerts S (2009). Goat Plaque or Peste des Petits Ruminants. *EAZWV Transmissible Disease Fact Sheet*.

[11] Chauhan HC, Chandel BS, Kher HN (2009). Peste des petits ruminants virus infection in animals. *Veterinary World*.

[12] Aruni AW, Lalitha PS, Mohan AC, Chitravelu P, Anbumani SP (1998). Histopathological study of a natural outbreak of Peste des petits ruminants in goats of Tamilnadu. *Small Ruminant Research*.

[13] Osman NA, Ali AS, Rahman ME, Fadol MA (2009). Antibody seroprevalences against Peste des Petits Ruminants (PPR) virus in sheep and goats in Sudan. *Tropical Animal Health and Production*.

[14] Sarker SC, Islam H (2001). Prevalence and risk factor assessment of Peste des petits ruminants in goats in Rajshahi, Bangladesh. *Veterinary World*.

[15] Kulkarni DD, Bhikane AU, Shaila MS, Varalakshmi P, Apte MP, Narladkar BW (1996). Peste des petits ruminants in goats in India. *Veterinary Record*.

[16] Hamdy FM, Dardiri AH, Nduaka O (1976). Etiology of the stomatitis pneumoenteritis complex in Nigerian dwarf goats. *Canadian Journal of Comparative Medicine*.

[17] Roeder PL, Obi TU (1999). Recognising peste de petits ruminants: a field manual. *FAO Animal Health Manual *.

[18] OIE (2012). *Peste des Petits Ruminants: Disease Factsheets*.

[19] Taylor WP, Al Busaidy S, Barrett T (1990). The epidemiology of peste des petits ruminants in the Sultanate of Oman. *Veterinary Microbiology*.

[20] Scot GR (1981). Rinderpest and peste des petits ruminants. *Virus Diseases of Food Animals*.

[21] Abraham G, Sintayehu A, Libeau G (2005). Antibody seroprevalences against peste des petits ruminants (PPR) virus in camels, cattle, goats and sheep in Ethiopia. *Preventive Veterinary Medicine*.

[22] NALEIC (2012). *Annual Report*.

[23] NALEIC (2010). *Peste des Petits Ruminants (PPR) Surveillance in Chama, Isoka, Lundazi, Mbala, Mpulungu and Nakonde Districts of Zambia*.

[24] Zepeda C World Organisation for Animal Health Seminar on Safeguarding Animal Health in Trade in the Caribbean.

[25] OIE (2010). *Manual of the Diagnostic Tests and Vaccines for Terrestrial Animals*.

[26] Karimuribo ED, Loomu PM, Mellau LSB (2011). Retrospective study on sero-epidemiology of peste des petits ruminants before its official confirmation in northern Tanzania in 2008. *Research Opinions in Animal and Veterinary Sciences*.

[27] Muse EA, Karimuribo ED, Gitao GC (2012). Epidemiological investigation into the introduction and factors for spread of Peste des Petits Ruminants, Southern Tanzania. *Onderstepoort Journal of Veterinary Research*.

[28] Ministry of Livestock and Fisheries Development Trade in Livestock between Zambia and Tanzania.

[29] Muse EA, Matondo RB, Karimuribo ED (2012). Clinico-pathological findings of the 2011 outbreak of Peste des Petits Ruminants (PPR) in Tandahimba district, Southern Tanzania. *Research Opinions in Animal and Veterinary Sciences*.

[30] Singh RP, Sreenivasa BP, Dhar P, Bandyopadhyay SK (2004). A sandwich-ELISA for the diagnosis of Peste des petits ruminants (PPR) infection in small ruminants using anti-nucleocapsid protein monoclonal antibody. *Archives of Virology*.

[31] Choi K-S, Nah J-J, Ko Y-J (2005). Rapid competitive enzyme-linked immunosorbent assay for detection of antibodies to peste des petits ruminants virus. *Clinical and Diagnostic Laboratory Immunology*.

[32] OIE (2009). *Peste des Petits Ruminants*.

[33] Radostits OM, Gay CC, Blood DC (2007). *Veterinary Medicine*.

